# REFRAME, REFORM, AND TRANSFORM: POLICY APPROACHES TO IMPROVE NURSING HOME QUALITY IN THE UNITED STATES

**DOI:** 10.1093/geroni/igac059.1562

**Published:** 2022-12-20

**Authors:** Angela Perone

**Affiliations:** University of California - Berkeley, Berkeley, California, United States

## Abstract

Nursing homes (NHs) are one of the most heavily regulated industries in the United States. However, concerns about quality of care remain, especially for more vulnerable residents, including those with dementia. Concerns only increased during recent natural disasters and the COVID-19 pandemic. Community and industry advocates have suggested diverse reforms that prompted new federal policies. This study poses the following research questions: (1) How do federal policies compare with proposals by community (staff and patient advocates) and industry (NH) advocates to improve quality of care in NHs before and during the pandemic; and (2) How do federal policies address micro, mezzo, and macro level issues? To address these questions, this study employs a multi-level comparison case study design. Primary data includes systematically collected and analyzed federal bills, CMS regulations, and community and industry reports, press releases, and website data from 2018 through 2022. Findings revealed that policy proposals fell into three categories: reframe, reform, and transform. Reframe approaches included minimal transformation at the micro-level. Reform approaches involved more mezzo and macro-level changes. Transform approaches proposed significant structural changes at the macro-level. Community advocates presented far more transformative changes than industry advocates and federal policymakers. Federal policies focused on reframe and reform solutions at the micro level and rarely proposed transforming macro-level structures (e.g., workforce structural inequities). This study has important implications for research, policy, and practice by exposing limitations and strengths of proposed solutions for addressing resident care and concludes with suggestions for better aligning with community advocates.

